# Impact of post-ablation pulmonary vein adhesions on robot-assisted thoracoscopic surgery lobectomy: a case report

**DOI:** 10.1186/s44215-025-00232-3

**Published:** 2025-11-14

**Authors:** Takashi Teishikata, Yusuke Okamoto, Masafumi Hiratsuka, Keiji Kamohara

**Affiliations:** Department of Thoracic and Cardiovascular Surgery, Faculty of Medicine, Saga University, 5-1-1 Nabeshima, Saga, 849-8501 Japan

**Keywords:** Robot-assisted thoracoscopic surgery, Atrial fibrillation, Ablation, Lung cancer

## Abstract

**Background:**

The prevalence of atrial fibrillation (AF) is increasing in Japan, largely because of the aging population. Catheter ablation, particularly pulmonary vein isolation, is a widely adopted intervention for maintaining sinus rhythm. We report a case of robot-assisted lobectomy for lung adenocarcinoma in a patient with a history of cryoablation for AF.

**Case presentation:**

A 76-year-old man with paroxysmal AF and prior cardiogenic stroke was referred for catheter ablation. Pre-ablation chest computed tomography revealed an enhancing 18 × 15 mm nodule in the right lower lobe, suggestive of lung cancer. The patient underwent cryoballoon ablation with successful pulmonary vein isolation. Three months later, a robot-assisted right lower lobectomy was performed. Intraoperatively, dense inflammatory adhesions were observed around the inferior pulmonary vein, likely induced by prior ablation, which significantly impeded dissection. The surgical technique was adapted accordingly, including the use of a 45-mm blue stapler owing to the increased tissue thickness. No adhesions were observed around the pulmonary artery or the bronchi. Lobectomy with lymph node dissection was performed without complications. The postoperative course was uneventful, and the patient was discharged on postoperative day 7.

**Conclusion:**

This case highlights the need for heightened intraoperative caution during lobectomy in patients with a history of catheter ablation. Ablation-induced adhesions around the pulmonary veins can obscure anatomical landmarks and complicate robot-assisted thoracic surgery, thereby increasing technical difficulty and potential procedural risks.

## Background

Owing to the aging population in Japan, the number of patients with atrial fibrillation (AF) is increasing [1]. Catheter ablation, mainly pulmonary vein isolation (PVI), is a common procedure for maintaining sinus rhythm. Pulmonary vein stenosis (PVS) is a serious complication of catheter ablation for AF, with a reported incidence ranging from 0 to 42% [2]. Ablation often involves cauterization of the left atrium near the pulmonary vein orifice, inducing an inflammatory response and subsequent fibrosis, which can result in PVS. Herein, we report a case of robot-assisted right lower lobectomy for lung cancer complicated by dense adhesions around the pulmonary vein, believed to result from recent cryoballoon ablation for AF.

## Case presentation

**Fig. 1:**
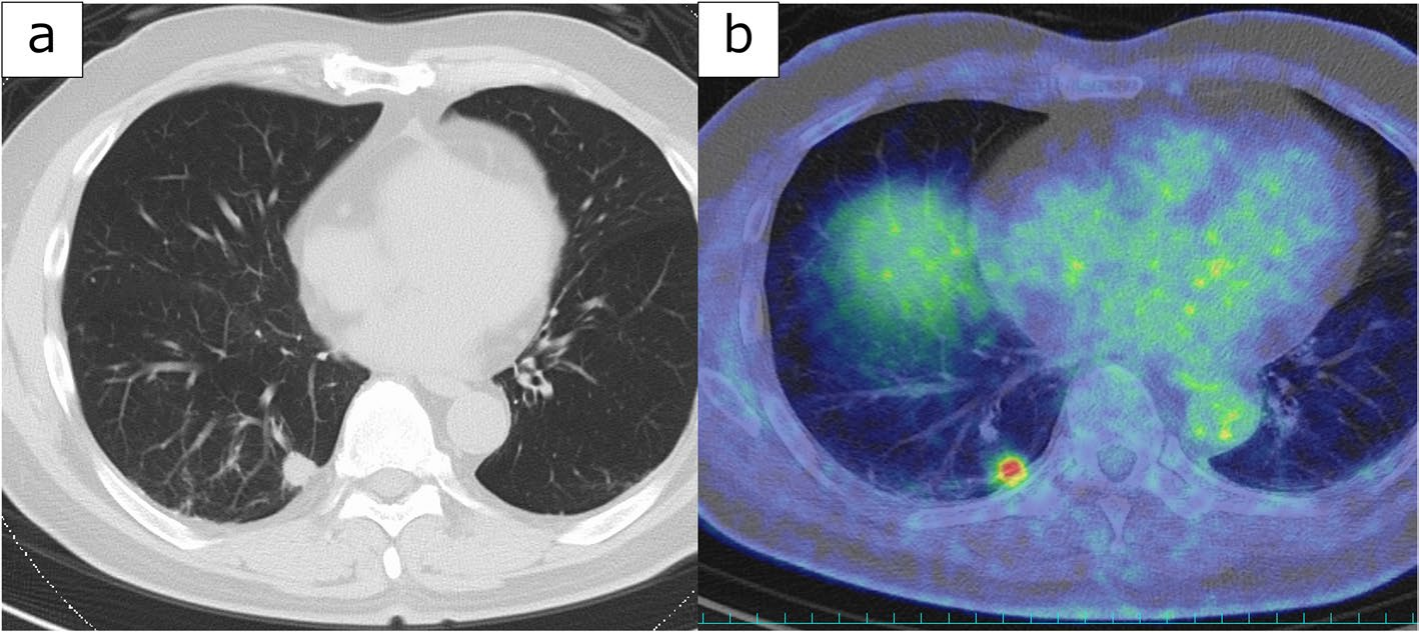
**a** Chest CT at initial presentation revealed an 18 × 15 mm nodule in the right lower lobe. **b** PET-CT showed high uptake in the nodule

**Fig. 2:**
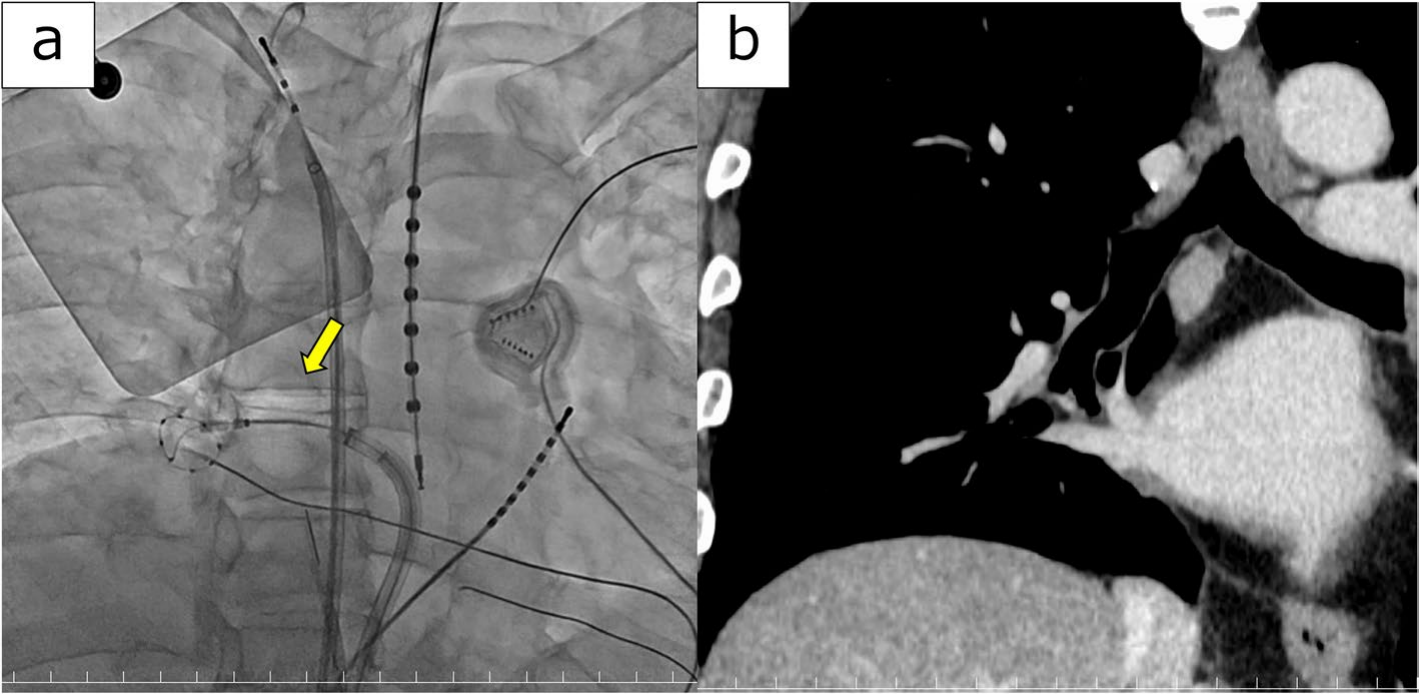
**a** Findings of cryoablation: A cryoballoon is positioned in the right inferior pulmonary vein (arrow). **b** Preoperative chest CT findings: No obvious abnormalities such as stenosis or wall thickening are observed in the inferior pulmonary vein

A 76-year-old man was referred to our hospital for ablation following paroxysmal AF and cardiogenic stroke. Pre-ablation chest computed tomography (CT) revealed an 18 × 15 mm contrast-enhanced subpleural nodule in the right lower lobe (Fig. 1a). Positron emission tomography-computed tomography showed high uptake, with a maximum standardized uptake value of 7.3, suggesting the possibility of primary lung cancer (Fig. 1b). Given the patient's recent stroke, management of AF was prioritized. Cryoballoon ablation for PVI was performed (Fig. 2a), after which the patient successfully returned to sinus rhythm. Robot-assisted thoracoscopic surgery (RATS) right lower lobectomy was planned for three months after ablation. Preoperative CT did not reveal any abnormal findings, such as stenosis or wall thickening, in the right inferior pulmonary vein (Fig. 2b).

During the surgical procedure, the patient was intubated and placed in the left lateral decubitus position. A 30° camera port was placed in the eighth intercostal space along the mid-axillary line. A posterior port was then placed in the same intercostal space 7 cm away from each camera port, and a second optional port was placed in the auricular triangle. An anterior port was placed in the sixth intercostal space along the anterior axillary line.

Initially, a partial resection of the nodule was performed. Intraoperative frozen-section pathological examination confirmed lung adenocarcinoma. Subsequently, right lower lobectomy with lymph node dissection was performed.

**Fig. 3:**
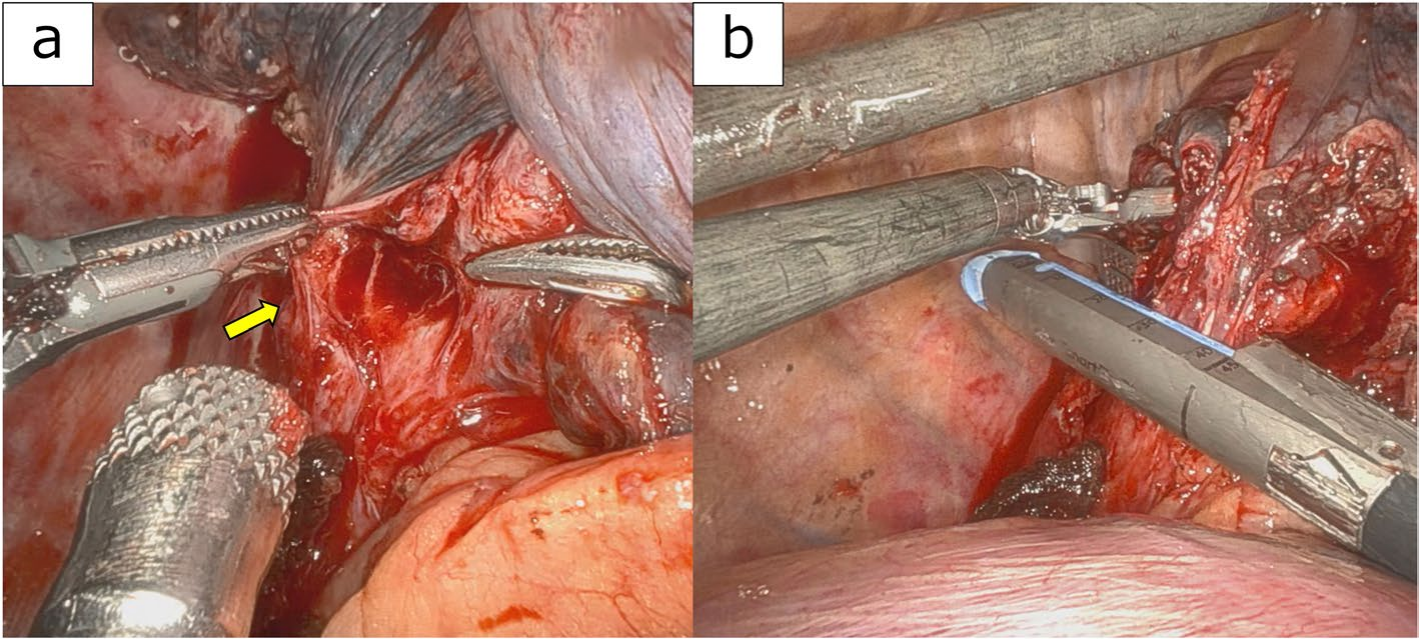
**a** Inflammatory adhesions observed around the right inferior pulmonary vein (arrow). **b** Thickened pulmonary vein transected using a SureForm Blue stapler

Dissection of the inferior pulmonary vein was difficult because of inflammatory-like adhesions (Fig. 3a). To avoid vascular injury, the adherent tissue was left on the vein stump. Consequently, the stump was thicker than usual, necessitating the use of a 45-mm blue cartridge (for thicker tissue) for transection instead of a standard white cartridge (for vascular structures) (Fig. 3b).

By contrast, no significant adhesions were observed around the pulmonary artery or bronchi. The surgery was completed with a lower lobectomy with lymph node dissection.

After RATS, the patient had an uneventful postoperative course and was discharged on postoperative day 7.

## Discussion and conclusions

Catheter ablation for AF primarily targets PVI, as ectopic triggers often arise from the antral regions surrounding the left superior, left inferior, right superior, and right inferior pulmonary veins [3]. After transseptal access to the left atrium, ablation lesions are delivered circumferentially around each pulmonary vein ostium, either point-by-point using radiofrequency energy or in a single application with cryoballoon technology [3]. Both strategies aim to achieve durable electrical isolation while preserving adjacent structures, and randomized trials have demonstrated comparable efficacy and safety between the two modalities [4].

PVS is a significant and potentially severe complication of AF ablation. Historically, PVS was primarily linked to radiofrequency ablation techniques; however, subsequent observations confirmed its occurrence following cryoablation as well [5]. Clinically, patients with PVS may exhibit a spectrum of symptoms, ranging from asymptomatic findings to severe dyspnea and pulmonary hypertension, often complicating the diagnosis because of the non-specificity of the presenting features [6]. Diagnostic challenges are compounded by the limitations of standard imaging modalities, although advanced imaging techniques, such as CT, magnetic resonance imaging, and ventilation-perfusion scans have been employed for accurate assessment [7].

In the present case, we encountered difficulty during RATS for lung cancer because of dense adhesions around the pulmonary veins, which complicated the dissection. Considering recent cryoablation, we speculate that these adhesions were the result of thermal injury and a subsequent inflammatory fibrotic response. Although PVS did not occur, a similar pathophysiological mechanism may have been involved. Notably, the peripheral side of the pulmonary vein could be dissected. According to the findings at the pulmonary vein transection site, inflammation was most pronounced at the pericardial sac and at the venous outlet. Considering the ablation target, inflammation would be expected to be more intense at the pericardial sac and pulmonary vein outlet. Therefore, in selected cases, segmentectomy, rather than lobectomy, may be technically feasible and safer.

The literature indicates that post-ablation pulmonary vein injury can lead to fibrosis and scarring, which sometimes progresses to stenosis or occlusion [8]. Such pathological changes can result in inflammatory responses and structural remodeling, making subsequent surgical interventions more challenging, as observed in our case. Moreover, in cases of complete pulmonary vein occlusion or necrosis, surgical resection, such as lobectomy, may be required. Histopathological examination during these procedures demonstrated significant inflammation and sclerosis around the affected veins [8, 9].

Although minimally invasive and robot-assisted techniques offer several advantages, they have some limitations. The absence of tactile feedback during robotic surgery can lead to excessive force application, potentially aggravating tissue damage or complicating dissection around scarred or adherent tissue [10, 11]. In our case, the absence of tactile feedback during robot-assisted surgery posed a technical challenge; therefore, we relied primarily on visual assessment rather than tactile sensation. Forcible dissection around the pulmonary vein carries the risk of vascular injury. In certain circumstances, conversion to video-assisted thoracoscopic surgery may provide a safer approach. Careful selection of the stapler is critical, as involvement of surrounding tissue can increase tissue thickness and the risk of staple malfunction. To the best of our knowledge, no previous reports have described RATS for lung cancer in patients with a history of AF ablation. Given the unique technical characteristics of robotic surgery, meticulous planning and intraoperative adaptability are essential to manage potential challenges. In patients with a history of AF ablation, adhesions around the pulmonary veins are not uncommon; therefore, tactile feedback in video-assisted thoracoscopic surgery or open thoracotomy, and visual cues in RATS, should guide safe dissection.

In conclusion, ablation-induced adhesions around the pulmonary veins can significantly complicate RATS procedures by obscuring the anatomy and increasing the risk of intraoperative complications, such as vascular injury. Therefore, surgeons operating on patients with a history of catheter ablation must anticipate these challenges and be prepared to adapt their surgical techniques to ensure safe and effective outcomes.

## Data Availability

The datasets used and/or analysed during the current study are available from the corresponding author on reasonable request.

## Abbreviations

AF: Atrial fibrillation
PVI: Pulmonary vein isolation
PVS: Pulmonary vein stenosis
RATS: Robot-assisted thoracoscopic surgery
CT: Computed tomography
